# Targeting specificity of APOBEC-based cytosine base editor in human iPSCs determined by whole genome sequencing

**DOI:** 10.1038/s41467-019-13342-8

**Published:** 2019-11-25

**Authors:** Erica McGrath, Hyunsu Shin, Linyi Zhang, Je-Nie Phue, Wells W. Wu, Rong-Fong Shen, Yoon-Young Jang, Javier Revollo, Zhaohui Ye

**Affiliations:** 1 Division of Cellular and Gene Therapies, Office of Tissues and Advanced Therapies, Center for Biologics Evaluation and Research, Food and Drug Administration, Silver Spring, MD USA; 2 Facility for Biotechnology Resources, Center for Biologics Evaluation and Research, Food and Drug Administration, Silver Spring, MD USA; 3 Department of Oncology, Johns Hopkins School of Medicine, Baltimore, MD USA; 4 Division of Genetic and Molecular Toxicology, National Center for Toxicology Research, Food and Drug Administration, Jefferson, AR USA

**Keywords:** CRISPR-Cas9 genome editing, Stem-cell research

## Abstract

DNA base editors have enabled genome editing without generating DNA double strand breaks. The applications of this technology have been reported in a variety of animal and plant systems, however, their editing specificity in human stem cells has not been studied by unbiased genome-wide analysis. Here we investigate the fidelity of cytidine deaminase-mediated base editing in human induced pluripotent stem cells (iPSCs) by whole genome sequencing after sustained or transient base editor expression. While base-edited iPSC clones without significant off-target modifications are identified, this study also reveals the potential of APOBEC-based base editors in inducing unintended point mutations outside of likely in silico-predicted CRISPR-Cas9 off-targets. The majority of the off-target mutations are C:G->T:A transitions or C:G->G:C transversions enriched for the APOBEC mutagenesis signature. These results demonstrate that cytosine base editor-mediated editing may result in unintended genetic modifications with distinct patterns from that of the conventional CRISPR-Cas nucleases.

## Abstract (web summary)

Cytidine base editors are powerful tools for making subtle genome alterations. Here the authors analyse edited human iPSCs with whole genome sequencing and reveal the spectrum of off-target effects.

## Introduction

Genome-editing tools, including engineered nucleases such as meganucleases, ZFNs, TALENs and CRISPR, are transforming medicine due to their unprecedented efficiency in altering genomic sequences in living cells. Recently, base editors have been developed by fusing catalytically impaired Cas proteins to DNA/RNA modifying enzymes such as nucleoside deaminases^1–4^. Base editors do not rely on generating DNA double-strand breaks to mediate genome editing, therefore potentially improving their safety profile by reducing the incidence of unwanted insertions, deletions and chromosomal rearrangement. Genome-editing specificities of base editors, particularly the adenine base editors, have been evaluated by various assays^5–8^. However, the mutagenic potential of APOBEC (apolipoprotein B mRNA editing catalytic polypeptide-like)-based cytosine base editors in clinically relevant cell types has not been evaluated by unbiased genome-wide analysis. Such analysis is important for demonstrating the safety profiles of these genome-editing tools, given the extensive evidence on the role of APOBEC family cytidine deaminases in causing mutations in human cancers^9–11^, as well as two recent reports of unintended mutations observed in mouse and plant systems^12,13^. In this study, the mutation landscapes of base-edited human induced pluripotent stem cells (iPSCs) were evaluated by whole-genome sequencing (WGS). We report that cytosine base editor could induce global unintended mutations enriched for C:G->T:A transitions and C:G->G:C transversions. Our results also suggest that these off-target mutations are likely caused by the base editor’s APOBEC activity that is not dependent on target DNA binding of Cas9.

## Results

### Effect of cytosine base editor long-term expression in iPSCs

**Fig. 1:**
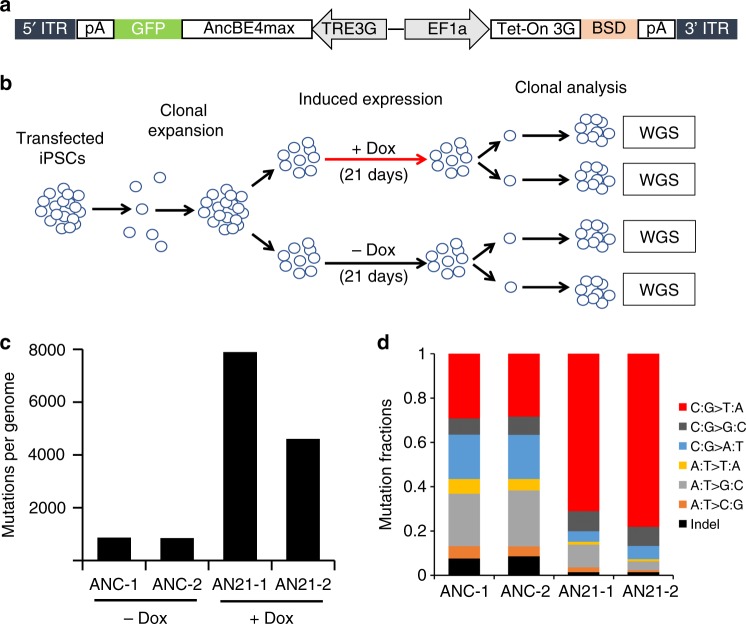
WGS analysis of iPSC clones after long-term cytidine base editor expression reveals increased mutations. **a** Schematic of the doxycycline-inducible XL-AncBE4max piggyBAC construct inserted into the CL1 line. **b** Diagrammatic representation of experimental design for clonal expansion and whole-genome sequencing analysis of iPSCs with or without doxycycline-induced 21-day base editor expression. **c** Mutation numbers in uninduced (ANC-1 and ANC-2) and induced (AN21-1 and AN21-2) iPSC clones. The numbers are total sequence variations, including indels and single nucleotide variations, as compared to the sequence of the parental CL1 iPSCs. **d** Fractions of each type of mutations in uninduced and induced clones. Source data for **c** and **d** are provided in the Source Data file.

Base editing was initially developed by fusing rat APOBEC1 to catalytically inactive CRISPR/Cas9^1^. Various further modifications have been made to enhance editing efficiencies and specificity, resulting in different versions of cytosine base editors^2–4,14–18^. The study reported here examined the specificity of the AncBE4max APOBEC1 variant, in an episomal vector-reprogrammed human iPSC line (Supplementary Fig. 1), because of its improved efficiency over previous versions of APOBEC1 editors^16^. In preliminary experiments, this editor more consistently achieved detectable editing events upon transient transfection of human iPSCs (Supplementary Fig. 2). Since base editor specificity is a particularly significant issue in cases where the editing components are continuously expressed, such as through viral vector-mediated editing or due to unintended integration of plasmid vectors, a doxycycline-inducible form of base editor (Fig. 1a) was first introduced into human iPSCs through piggyBAC transposon integration to study the effect of long-term base editor expression. To minimize the interference of background mutations, acquired by individual iPSCs during culture maintenance, a clone of the blasticidin-resistant doxycycline-inducible iPSCs was used for base editor induction experiments (Fig. 1b). iPSCs carrying the base editor expression cassette were expanded in the absence or presence of doxycycline for 3 weeks before individual clones were isolated and further expanded, in the absence of doxycycline, for genomic DNA isolation and WGS (Fig. 1b The sequencing results from all the iPSC clones were compared to those obtained from the parental iPSCs to obtain the numbers of sequence variants (Table 1, Supplementary Table 1 and Supplementary Data 1). While 847 and 869 sequence variations were detected in the two clones derived from the non-induced group, significantly higher numbers of mutations (7896 and 4605) were detected in the two clones that had been treated with doxycycline for 3 weeks (Fig. 1c). The increase of mutation frequencies in the dox-treated samples cannot be explained solely by differences in spontaneous mutations associated with clonal selection at the end of doxycycline treatment, as the numbers greatly exceeded that of the estimated spontaneous mutations (3–30 mutations per haploid genome per mitotic division in somatic cells) after a 3-week expansion of human iPSCs (~20 cell divisions)^19–21^. To gain more insight into the nature of these increased mutation rates and whether they were related to the cytosine base editor, the mutation spectrum in each clone was analyzed. While the single nucleotide substitutions in the two control clones without doxycycline induction revealed an even distribution of transition and transversion types, significantly more C:G to T:A transitions were observed in the two clones that have had long-term base editor expression (Fig. 1d).

**Table 1:** Summary of whole-genome sequencing of iPSC clones.

Duration of editor expression	iPSC clone	Editing target	Form of editor expression	Total variations	C:G > T:A	C:G > G:C	C:G > A:T	A:T > C:G	A:T > G:C	A:T > T:A	Indel	Exonic	Non-syn^e^	Non-syn C>T & C>G^f^
Long-term	ANC-1	–	Integrated (−Dox)	869	254	63	175	48	206	57	66	8	4	2
	ANC-2			847	241	69	169	38	214	43	73	6	4	3
	AN21-1		Integrated (+Dox)	7896	5612	718	371	167	817	98	113	105	67	50
	AN21-2			4605	3597	399	275	40	185	46	63	41	28	28
Transient transfection	N1^a^	–	– (Control Plasmid)	186	46	6	53	14	20	3	44	2	2	0
	N2^a^			171	42	3	66	5	12	4	39	3	2	1
	N3^a^			145	39	4	36	7	12	6	41	2	2	1
	HK31^b^	HEK3	Plasmid	164	55	14	41	6	16	5	27	1	0	0
	HK34^b^			2300	2086	59	71	10	23	6	45	29	20	19
	HK36^b^			235	83	33	47	5	20	10	37	3	3	3
	HK32M^c^		RNA	242	96	16	59	9	11	9	39	4	2	1
	HK33M^c^			836	561	144	75	7	15	5	29	12	7	6
	HK34M^c^			113	40	5	30	2	7	3	26	0	0	0
	EX1M	EXM1	RNA	272	121	17	66	7	14	12	36	3	1	1
	RF23M^d^	RNF2	RNA	599	437	41	69	5	9	12	26	5	2	2
	RF24M^d^			1813	1573	96	76	13	15	7	33	22	14	12

Please see Supplementary Data 1 for a complete annotation of all the mutations

^a,b,c,d^Clones with the same letter note were isolated from the same transfection reaction (e.g. HK31, HK34 and HK36 are individual clones from the same transfection)

^e^Including non-synonym and stop-gain mutations that are located in exonic regions

^f^C:G > T:A and C:G > G:C mutations among non-synonym and stop-gain mutations that are located in exonic regions

### Mutation frequency after base editor transient transfection

**Fig. 2:**
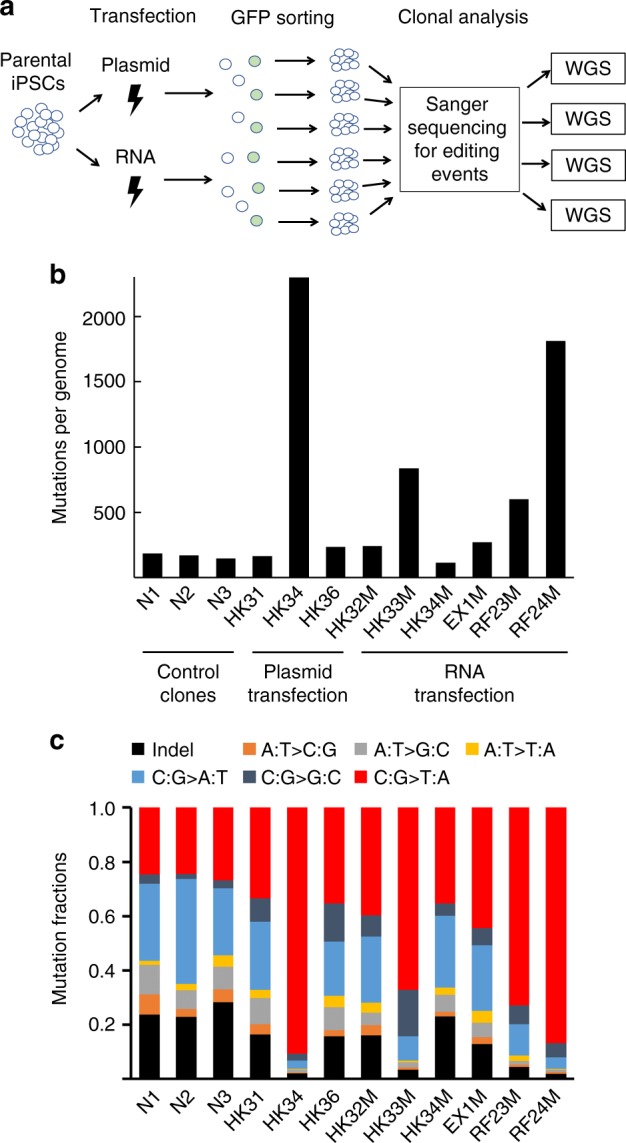
Mutation frequencies in iPSC clones base-edited by transient transfection. **a** Diagrammatic representation of experimental design for whole-genome sequencing analysis of iPSCs edited at the HEK3, RNF2 or EMX1 locus after transient transfection with either the plasmid or the RNA form of base editor. Clones transfected by a GFP-only plasmid vector were used as procedure controls for the analysis. **b** Mutation numbers in control iPSC clones and base-edited iPSC clones after plasmid or RNA transfection. **c** Fractions of mutations in control and base-edited iPSC clones. Source data for **b** and **c** are provided in the Source Data file.

The long-term expression experiment examines the editing tool’s potential risks in a worst-case scenario. Because it is also important to determine the impact of base editing on genomic DNA integrity in a more clinically relevant procedure, experiments were performed to edit iPSCs by transient transfection of base-editing reagents (Fig. 2a). The pCMV-AncBE4max-GFP plasmid vector and guide RNA expressing plasmids were used for transfection. Cells that had been successfully transfected were enriched by flow sorting for GFP expression^16^. To further reduce the probability of base editor random integration in the genome, editing experiments were also conducted using in vitro transcribed mRNA encoding the AncBE4max editor together with chemically synthesized sgRNA targeting the HEK3, EMX1 or RNF2 locus. GFP+ cells were flow-sorted and plated for clonal expansion followed by Sanger sequencing to determine the on-target editing event. iPSC clones with confirmed on-target editing were then subjected to WGS analysis. Three iPSC clones transfected by the pmaxGFP control plasmid were included as procedure controls to determine the background level of mutations occurring during transfection and clonal expansion (Fig. 2a).

In transiently transfected iPSCs, comparable numbers of mutations (range 145–186) were detected in the three control clones when compared to the parental iPSCs (Fig. 2b). Among these mutations, ~25% of them in each clone are C:G->T:A transitions (Table 1 and Fig. 2c). In contrast, the number of mutations varied significantly among the nine transiently transfected base-edited iPSC clones, ranging in frequencies from below those observed in the control cells (113) to frequencies that were more than tenfold higher (2300) (Table 1 and Fig. 2b). Of note, the significantly increased numbers of mutations were observed in both the plasmid-transfected and the RNA-transfected clones. Meanwhile, clones with mutation numbers comparable to the controls were also identified in both groups. The mutation spectrum in each clone also varied with the percentage of C:G->T:A transitions increasing with the total number of mutations detected, reaching 86.7% and 90.7%, respectively, in the two clones with the highest number of mutations (1813 and 2300, respectively) (Table 1 and Fig. 2c).

### Mutations are enriched for the APOBEC mutagenesis signature

**Fig. 3:**
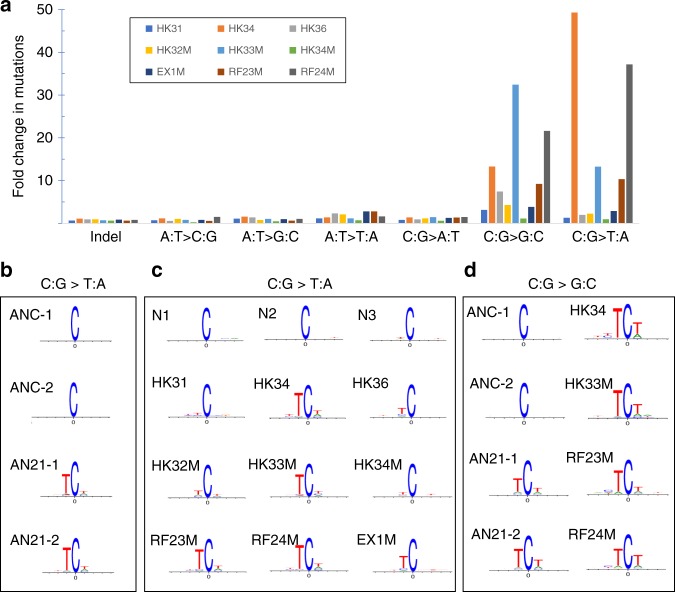
Characterizations of the unintended mutations and identification of a sequence signature related to APOBEC activity. **a** Fold change of each type of mutation in base-edited iPSC clones as compared to control clones. A fold change is calculated by dividing the number of one type of mutation detected in one clone by the average number of that mutation type (indicated on x-axis) detected in three control clones N1, N2 and N3. Source data are provided in the Source Data file. **b** Sequence logos of the conserved bases around the C>T mutations in each iPSC clone after inducible base editor expression. The mutated C is shown at position 0. The overall height of each stack indicates the sequence conservation at that position, while the height of symbols within the stack indicates the relative frequency of each nucleic acid at that position^22^. Sequence logos from position −5 to position 25 are shown in Supplementary Fig. 3. **c** Sequence logos of the conserved bases around the C>T mutations in iPSC clones edited by transient transfection. **d** Sequence logos of the conserved bases around the C->G mutations in iPSC clones that each has more than 40 detected C:G->G:C transversions.

In addition to the C:G->T:A transitions, another mutation type with a notable increase in the edited cells as compared to controls clones is the C:G->G:C transversion, even though their absolute numbers are significantly fewer than that of C:G->T:A transition (Fig. 3a). Having determined that C:G->T:A transitions constitute the majority of the mutations detected in iPSC clones analyzed, potential sequence conservations at positions near the C-to-T transitions were further analyzed^22^. A preference for a TCW (C is where the mutation occurs; W = T or A) motif was evident in both the stably transfected iPSC clones after 21-day dox treatment (Fig. 3b) and in all the transiently transfected clones with high mutation loads (Fig. 3c), while no dominant local sequence context pattern was detected in control clones or base-edited clones with low mutation loads (Fig. 3 and Supplementary Fig. 3). The same analysis, performed on C-to-G transversions in iPSC clones that have more than 40 mutations of this type, also identified this TCW motif (Fig. 3d). The observed enrichment in TCW->TTW or TCW->TGW mutations has been previously reported as the APOBEC mutagenesis signature in studies of several types of human cancers^9–11^. These results suggest a contribution of the APOBEC-based cytosine base editor to the overall increase of single nucleotide substitutions observed in the iPSCs.

### Mutations are random and outside of CRISPR–Cas9 off-targets

**Fig. 4:**
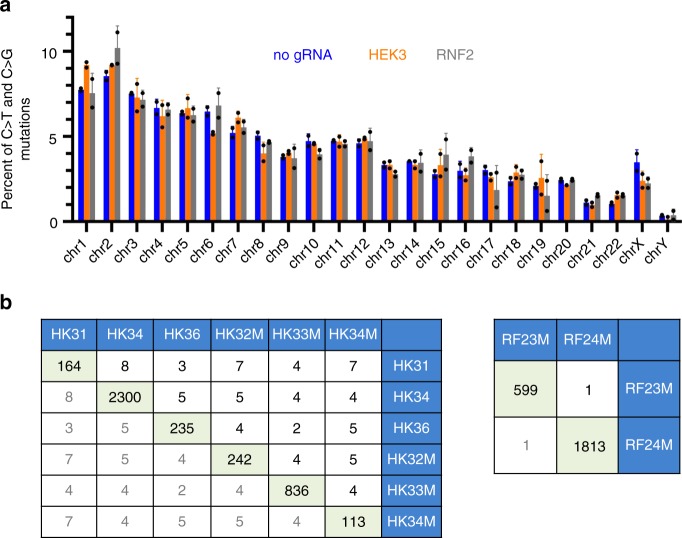
The off-target mutations are randomly distributed. **a** Chromosomal distribution of C:G>T:A and C:G>G:C mutations in iPSC clones that each has >500 total mutations. Error bar represents standard deviation (*n* = 2). Source data are provided in the Source Data file. **b** Numbers of mutation overlaps between iPSC clones edited at either HEK3 or RNF2 locus. The numbers of mutations include on-target mutation(s). A complete comparison of mutations among all sequenced iPSC clones is shown in Supplementary Table 4.

In addition to analyzing the local sequence context preference of the mutations, additional analyses were conducted to determine whether location preference exists for these unintended mutations. In the long-term expression experiments involving stably transfected iPSCs, base editor expression was induced in the absence of any exogenous gRNA, indicating that gRNA-mediated stable Cas9–DNA binding is not required for the observed mutagenesis effect. Comparable chromosomal distribution of mutations was also observed among iPSCs treated with base editor in the absence or presence of a guide RNA (Fig. 4a). To further address this issue, in silico-predicted likely CRISPR/Cas9 off-targets of gRNA targeting the HEK3, RNF2 or EMX1 locus were analyzed in the transiently transfected base-edited iPSC clones. No sequence variation was observed at any of the 557 most likely potential off-target sites, each of which has four or fewer nucleotide mismatches to the gRNA target and some contain the TCW sequence motif (Supplementary Data 2), indicating that the observed APOBEC-like mutagenesis events do not occur preferentially at potential CRISPR/Cas9 off-target sites. Additional analyses on 15–20 bases downstream of all the mutated cytidines in three clones containing the highest numbers of mutations (AN21–2, HK34 and RF24M) showed that the frequencies of those six bases containing at least one CRISPR/Cas9 PAM site (NGG) were 0.184, 0.185 and 0.204, respectively. This is comparable to the frequency (0.188) of a random set of one million 6-base sequences in human genome (hg38), suggesting a majority of the off-target mutations are PAM-independent.

To determine whether APOBEC mutation hotspots exist in the human genome, locations of all sequence variants in each iPSC clone were compared to those of the other clones in order to estimate the extent of recurrent mutations. Strikingly, there were very few overlaps among the mutations from different clones (Supplementary Table 2 and Fig. 4b). The exceptions are the four clones (ANC-1, ANC-2, AN21–1, AN21–2) from the long-term expression experiment, among which between 400 and 500 mutations are shared. These shared mutations, however, are most likely the pre-existing ones in the starting iPSC clone that was used for the doxycycline induction experiment. As shown in Fig. 1, a single blasticidin-resistant clone was selected to initiate the long-term expression experiment to ensure that the cells treated with and without Dox have minimal background genetic variations to start with. The relatively more even distribution of mutation types (Fig. 1d) and a lack of pattern in local sequence context around mutated cytosines (Fig. 3b) further support that these mutations were carried over from the parental clone. These results demonstrate that the APOBEC mutagenesis caused by the base editor is random without apparent genomic hotspots.

## Discussion

Together with recent studies involving BE3 editor specificity in mouse and plant systems^12,13^, the current study on AncBE4max in clinically relevant human iPSCs raises the issue that APOBEC activity of cytidine base editors can potentially result in unintended point mutations in human cells. Compared to the BE3 used in the previous reports, the AncBE4max used in this study is a further engineered version and has been shown to have higher on-target editing efficiency^16^. To date, various forms of cytosine base editors have been created by fusing catalytically impaired CRISPR proteins with different molecules that contain cytosine deaminase activity^23–31^. It is reasonable to speculate that the off-target mutagenic effects of these cytosine base editors may differ, both in the rate and the molecular spectrum. Future studies aimed at understanding the relative efficiency and specificity of each base-editing tool will likely benefit potential clinical applications of this technology.

Findings in this study also have implications for selecting base editor delivery vehicles in therapeutic development. Viral vectors such as those derived from adeno-associated viruses (AAVs) remain popular choices for in vivo delivery of genome-editing agents. The viral vector approach, however, may result in a more sustained presence of editing enzymatic activity in the cells, which represents a potential safety concern as higher mutation loads were observed in the base editor long-term expression experiment in this study (Fig. 1). Incidental integration of transiently transfected plasmids during ex vivo genetic modification could also result in sustained editor expression; this low-probability but potentially high-impact event should be considered in planning risk assessment of relevant clinical research.

Data from this study and the previous reports do not definitively prove which module(s) of the base editors are responsible for the mutagenesis activity; however, the enrichment of the TCW nucleotide motif among the detected mutation sites strongly suggests that the APOBEC activity is the cause of the observed increase in mutations. As an additional control, we re-analyzed WGS data from two previously reported CRISPR/Cas9-edited iPSC clones^32^, and found no conserved pattern of local sequence context for mutated cytosine or guanine residues among the sites of single nucleotide variations (Supplementary Fig. 4). This analysis, along with the observation that these mutations do not correlate with in silico predicted Cas9/gRNA off-targets, suggests that ectopic expression of Cas9 or Cas9-D10A in human iPSCs is not likely to contribute to the mutation signature observed in this study. Although the APOBEC mutagenesis signature was originally proposed based on correlation analyses of the mutation signature and tumor APOBEC expression levels^9–11^, the potential role of APOBEC proteins in mutagenesis is further supported by data from in vitro experiments including those conducted in an *Escherichia coli* assay and those performed in DT40- and 293-based cellular systems^33–35^. It should be noted that the base editor used in this study has been further engineered and contains an APOBEC domain that is different from the naturally occurring forms of APOBECs^16^. Future definitive studies on the contribution of this engineered deaminase domain to the off-target mutations could facilitate efforts to further improve base editor fidelity.

Considerable intra-experiment variability was observed in terms of mutation loads among clones edited by transient base editor expression. For example, HK31, HK34 and HK36 were isolated from a single transfection but vary significantly in mutation numbers. Likewise, the three RNA-transfected clones HK32M, HK33M and HK34M were also isolated from a single transfection reaction and have wide-ranging mutation loads (Table 1). The cause to such variability is intrinsic to the transfection technology; specifically it is likely the result of varying amount of nucleic acids materials (e.g. plasmid DNA or mRNA encoding base editor) delivered to each individual cell during a transfection reaction. The number of off-target mutations in a clone is likely to correlate with, although may not be proportional to, the amount of base editor molecules the cell received. This study has identified a few base-edited iPSC clones, such as HK31 (involving plasmid transfection) and HK34M (involving RNA transfection), that contained nearly comparable numbers of mutations to the control clones with no nonsynonymous sequence changes in exonic regions (Table 1). We hypothesize that these clones with lower mutation loads have had base editor expression at levels that are sufficient for mediating on-target modification yet not as high or sustaining as that in some other clones (e.g. HK34 and HK33M) that resulted in substantial off-target mutations. This observation suggests the importance for future studies to evaluate the relationship between base editor expression level and off-target effect. Identifying the appropriate base editor expression levels, and feasible approaches to control them, may be crucial for achieving optimal editing efficiency and specificity. It should be noted that because these off-target mutations are random and unlikely to be predicted by current in silico programs that were designed to predict CRISPR off targets (Supplementary Data 2 and Supplementary Table 2), outcome of base editing should be determined by unbiased sequencing approaches.

While not addressed in this study, the fact that APOBEC1 is best characterize for its function in editing Apolipoprotein B RNA sequence raised the concern that unintended RNA editing could occur during the genomic base-editing process. Several recent reports have not only confirmed that both cytosine base editors and adenine base editors could result in widespread RNA editing, but also described the initial efforts to reduce such RNA off-target activities by protein engineering^36–39^. Similar approaches aimed at reducing DNA off-target activities could further improve safety of this technology.

## Methods

### iPSC generation and characterization

Experiments using human iPSC lines were conducted in accordance with Johns Hopkins Institutional Stem Cell Research Oversight Committee regulations following protocols approved by the Johns Hopkins IRB and the U.S. Food and Drug Administration IRB. The human iPSC line CL1 was generated from mobilized peripheral blood CD34+ cells by transient transfection of episomal plasmids^40^. Briefly, CD34+ cells from a healthy male donor were purchased from AllCells (Alameda, CA, USA) and were cultured in StemSpan medium (STEMCELL Technologies, Vancouver, BC, Canada) supplemented with stem cell factor (SCF), flt3-ligand (FL) and thrombopoietin (TPO) (Peprotech, Rocky Hill, NJ, USA) for 4 days before being reprogrammed by an episomal plasmid pEB-C5 expressing five transcription factors (Oct4, Sox2, Klf4, c-Myc and Lin28) using a Nucleofector II device (Lonza, Walkersville, MD, USA). The transfected cells were plated onto irradiated human feeder cells TW3R^41^ with a human ESC culture medium that consisted of KNOCKOUT DMEM supplemented with 20% KNOCKOUT Serum Replacement (KSR), 2 mM l-glutamine, 2 mM nonessential amino acids, 1× antibiotic/antimycotic mix (all these medium components were from Invitrogen, Carlsbad, CA, USA), 0.1 mM β-mercaptoethanol (Sigma, St. Louis, MO, USA), and 10 ng/mL bFGF (Peprotech, Rocky Hill, NJ, USA). At day 15 of reprogramming, TRA-1–60 fluorescent staining (1:50; Miltenyi Biotech, Bergisch Gladbach, Germany, catalog no. 130–122–965) was performed, and the positive colonies were manually picked and expanded on human feeder cells for eight passages. The established CL1 iPSC line was then adapted and maintained in STEMMACS iPS-Brew medium (Miltenyi Biotech, Bergisch Gladbach, Germany) on vitronectin-coated plates. Accutase (MilliporeSigma, Burlington, MA, USA) was used to dissociate the cells when passaging. Rho-associated kinase (ROCK) inhibitor Y27632 at 10 μM was added to the medium for the first 18–24 h at each passage. Karyotyping of CL1 cell line at passage 12 was performed by WiCell Cytogenetics Lab (Madison, WI, USA). Short tandem repeat (STR) Profiling was performed by Johns Hopkins University Genetic Resources Core Facility (Baltimore, MD, USA). iPSC characterization is summarized in Supplementary Fig. 1.

For characterization of CL1 for pluripotency-related marker expression, cells were grown on vitronectin-coated plates. Prior to imunocytochemistry staining for OCT4 and NANOG, cells were fixed with 4% paraformaldehyde and washed three times with cold PBS. Fixed cells were incubated with mouse anti-OCT4 (EMD Millipore, Burlington, MA, USA, catalog no. MAB4401) or anti-NANOG (BD Pharmingen, Franklin Lakes, NJ, USA, catalog no. 560109) antibodies in PBS (1:500) for 1 h at room temperature and washed three times with cold PBS. Next, cells were incubated with Alexa Fluor 488 secondary antibody (Invitrogen, Carlsbad, CA, USA, catalog no. A11001) in PBS (1:500) for 1 h at room temperature. After secondary antibody incubation, cells were washed and counterstained with DAPI (1:5000; Sigma, St. Louis, MO, USA) before immunofluorescence analysis. Images were taken with a Life Technologies EVOS FL Auto fluorescent microscope at 20× magnification. Scale bars represent 100 µm. For SSEA3 and TRA-1–60 flow cytometry analysis, cells were digested with Accutase and washed with PBS. A total of 1 × 10^6^ cells were incubated with Alexa 488 SSEA3 conjugate antibody (1:20; BioLegend, San Diego, USA, catalog no. 330306) for 30 min at 4 °C, washed in PBS, then analyzed using a BD LRS Fortessa flow cytometer. 1 × 10^6^ cells were incubated with anit-TRA-1–60 primary antibody (1:200; MilliporeSigma, Burlington, MA, USA, catalog no. MAB4360) for 30 min at 4 °C, washed with PBS, then incubated with appropriate Alexa Fluor 555 secondary antibody (1:500; Invitrogen, Carlsbad, CA, USA, catalog no. A21426) for 30 min at 4 °C. Cells were washed with PBS, then analyzed using a BD LRS Fortessa flow cytometer.

Teratoma formation experiments were approved by IACUC at Johns Hopkins University School of Medicine. Care of all experimental animals was in accordance with institutional guidelines. The iPSCs (5 × 10^6^) were harvested by cell scraper and were mixed with Matrigel (BD Biosciences, 5 mg/mL) in a final volume of 200 μL, and intramuscularly injected into the hind limbs of 8-week-old NSG mice (obtained from Jackson Laboratory). Mice were sacrificed 8 weeks after injection for teratomas harvest. The tumor tissue was fixed in 10% buffered formalin and then examined by a routine wax-embedding histological procedure. The paraffin sections were stained with hematoxylin and eosin. The typical morphologies of endoderm, mesoderm and ectoderm were observed under microscope.

### Construction of an inducible base editor expressing vector

A piggyBAC doxycycline-inducible vector XLone-GFP was used for cloning the base editor gene. XLone-GFP was a gift from Xiaojun Lian (Addgene plasmid no. 96930; http://n2t.net/addgene:96930; RRID:Addgene_96930). XL-AncBE4max was generated through Gibson Assembly (New England Biolabs, Ipswich, MA, USA) by combining a SpeI- and KpnI-digested XLone-GFP backbone with double-stranded PCR amplified AncBE4max-GFP fragment. Sequences of PCR primers used for amplifying the AncBE4max-GFP transgene are listed in Supplementary Table 3.

### Inducible base editor expression in human iPSCs

CL1 iPSCs (at passage 19) were dissociated with Accutase, and 1 × 10^6^ cells were transfected with 5 μg of PB-XL-AncBE4max transposon and 5 μg of piggyBac transposase plasmid^42^ using P3 Primary Cell buffer and the hES H9 program on a 4D-Nucleofector (Lonza Walkersville). The transfected cells were plated onto vitronectin-coated plate with iPS-Brew medium in the presence of 10 μM ROCK inhibitor Y27632. Two days after plating, blasticidin was added to the medium at 10 μg/mL for 1 week to select stably transfected cells. Single cell suspension was then plated into vitronectin-coated 96-well plates to obtain subclones of the stably transfected cells. Cells from one subclone were split into two culture conditions. To one of the subculture conditions, doxycycline (1 μg/mL) was added. Cells were maintained for 21 days either with or without daily doxycycline addition. During this period of expansion, cells from the two conditions were always passaged at the same time and were adjusted to maintain the same initial plating cell density (equivalent to 2 × 10^5^ per well in a 6-well plate) at each passage. After 21 days, single cell-derived clones were again expanded, without doxycycline, from each condition for about 14 more days before genomic DNA isolation and sequencing.

### iPSC base editing by transient transfection

The pCMV_AncBE4max_P2A_GFP plasmid was a gift from David Liu (Addgene plasmid no. 112100; http://n2t.net/addgene:112100; RRID:Addgene_112100). mRNA was in vitro transcribed from pCMV_AncBE4max_P2A_GFP plasmid using mMESSAGE mMACHINE T7 ULTRA Transcription Kit (Invitrogen) according to the manufacturer’s manual. Synthetic sgRNA targeting HEK3 (GGCCCAGACTGAGCACGTGA), RNF2 (GTCATCTTAGTCATTACCTG) and EMX1 (GAGTCCGAGCAGAAGAAGAA) were purchased from Synthego (Menlo Park, CA, USA).

iPSCs at passages between 19–21 were harvested by Accutase and washed with PBS before transfection. For plasmid-mediated editing, 1 × 10^6^ cells were resuspended in 100 μL of P3 Primary Cell buffer with 9 μg of pCMC_AncBE4max_P2A_GFP and 3 μg of gRNA-expressing plasmid, before transfection with a 4D-Nucleofector using the built-in hES H9 program. Alternatively, 1 × 10^6^ iPSCs were resuspended in 100 μL of P3 Primary Cell buffer with 3 μg of in vitro transcribed AncBE4max mRNA and 90 pmol of synthetic sgRNA before nucleofection. The transected cells were plated onto vitronectin-coated plate in iPSC-Brew medium with ROCK inhibitor for 2 days before GFP sorting for clonal expansion and screening. Genomic DNA was isolated using Quick-DNA Miniprep Kit (Zymo Research) from sorted GFP+ cell-expanded (10–16 days) clones and was used as template for PCR amplification of the targeted region. Sequences of PCR primers used for amplifying the target regions can be found in Supplementary Table 3. Sanger sequencing of the amplicons was performed to identify clones that have the desired base-editing outcomes.

### WGS and bioinformatics

Genomic DNA from each iPSC clone was isolated using Quick-DNA Miniprep Kit (Zymo Research, Irvine, CA, USA). Library preparation and sequencing were performed on the Illumina platforms by GENEWIZ (South Plainfield, NJ, USA). NEBNext Ultra II DNA Library Prep Kit for Illumina was used following the manufacturer’s recommendations. The genomic DNA was fragmented by acoustic shearing with a Covaris S220 instrument. Fragmented DNA was cleaned up and end repaired. Adapters were ligated after adenylation of the 3′ ends followed by enrichment by limited cycle PCR. DNA libraries were validated using a DNA 1000 Chip on the Agilent 2100 Bioanalyzer (Agilent Technologies, Palo Alto, CA, USA), and quantified using Qubit 2.0 Fluorometer. The DNA libraries were also quantified by real-time PCR (Applied Biosystems, Carlsbad, CA, USA), clustered on two lanes of a flowcell, and loaded on the Illumina HiSeq instrument according to manufacturer’s instructions. The samples were sequenced using a 2 × 150 paired-end (PE) configuration. Image analysis and base calling were conducted by the HiSeq Control Software (HCS) on the HiSeq instrument.

For analyses of the sequencing data, BWA 0.7.17-r1188, Samtools 1.9, Picardtools, Varscan 2.4.3 and Weblogo 3.6.0 were downloaded from the Bioconda repository (https://bioconda.github.io/). Resulting fastq files were aligned by BWA mem to the UCSC hg38 human genome (available at https://support.illumina.com/sequencing/sequencing_software/igenome.html). Alignments were merged and sorted by Samtools, and duplicate reads were marked by Picardtools. Whole-genome sequencing and alignment statistics were obtained by the “CollectRawWgsMetrics” and “samtools depth” algorithms from Picardtools and Samtools, respectively. Mutations were called by Varscan somatic by comparing the reads of each clone to the reads of the parental clone. Somatic mutations with *p* < 0.001, “variant allele frequency in normal” < 5%, “variant allele frequency in tumor” > 33.33%, and “reads supporting variant in tumor” > 6 were considered legitimate calls. These filtering criteria has previously been shown to accurately reflect somatic mutation loads in mammalian genomes^43^. Sequence consensus for each sample were created by Weblogo by generating FASTAs containing the adjacent bases of their C:G>T:A mutations. A summary of WGS statistics can be found in Supplementary Table 1. Mutations were annotated by wAnnovar (http://wannovar.wglab.org/) and are available as Supplementary Data 1.

The list of potential off-target sites, shown in Supplementary Data 2, of the HEK3, EMX1 and RNF2 gRNAs was generated by combining the sequences reported in a previous study^1^ and sequences predicted by WTSI Genome Editing (WGE, https://www.sanger.ac.uk/htgt/wge/)^44^, CHOPCHOP (https://chopchop.cbu.uib.no/)^45,46^, Cas-OFFinder (http://www.rgenome.net/cas-offinder/)^47^, CRISPOR (http://crispor.tefor.net/)^48^. The off-targets presented in Supplementary Data 2 are listed based on the Cutting Frequency Determination (CFD) score starting from the highest.

### Validation of mutations identified by WGS

Two approaches were used to validate the results of WGS analysis. In the amplicon sequencing approach, 62 mutations identified by WGS in clones AN21-1, HK34M and RF24M were analyzed. PCRs were performed with the Evrogen Encyclo Plus PCR kit on a thermocycler with the following temperature profile: 95 °C × 3 min + (95 °C × 30 s + 56 °C × 30 s + 72 °C × 30 s) × 40 + 10 °C hold. Each reaction contained 0.25 μM of each forward and reverse oligos and 2.5 ng of template DNA. Products were diluted in water to 0.5 ng/μL and miniaturized Nextera sequencing libraries were prepared in-parallel in a 96-well plate according to manufacturer’s instruction^49^. The library was paired-end sequenced (75 × 2) sequenced on NextSeq500 instrument (the sequencer and kit were from Illumina). Resulting fastq files were paired-end aligned by the “bwa mem” algorithm from BWA to the UCSC hg38 human genome and sorted by Samtools. PCR allele frequency (PCR:AF) calls were performed by Varscan’s pileup2snp on reads with mapping quality ≥30, on *Q* ≥ 20 bases, and on the 500-bp region spanning the corresponding PCR target. Depths (PCR:DP) were calculated by Samtools on reads with mapping quality ≥30, on *Q* ≥ 20 bases, and on the 1-bp corresponding to the potential mutation for each PCR target. Mutations in 59 sites (out of the 62 sites analyzed) were confirmed (Supplementary Data 3).

In addition, Sanger sequencing approach was also used to validate the 24 nonsynonymous exonic single nucleotide variations in clone AN21-2. The genomic regions of these mutation sites were amplified by PCR using Q5® High-Fidelity DNA Polymerase (NEB, Ipswich, MA, USA). PCR products were purified using the DNA Clean & Concentrator Kit (Zymo Research, Irvine, CA, USA) and sequenced by capillary electrophoresis. Mutation status in all 24 sites were confirmed (Supplementary Fig. 5).

### Reporting summary

Further information on research design is available in the Nature Research Reporting Summary linked to this article.

## Supplementary information

Supplementary Information

Description of Additional Supplementary Files

Supplementary Data 1

Supplementary Data 2

Supplementary Data 3

Reporting Summary

## Data Availability

Sequence data that support the findings of this study have been deposited in Sequence Read Archive (SRA) with the following accession code “SRP221668”. The source data underlying Figs. 1c, d, 2b, c, 3a and 4a are provided as a Source Data file. All other data are available from the authors upon reasonable request. All plasmids used in this study are available from Addgene: PB-XL-AncBE4max (#136254), gRNA-HEK3-Puro (#136282), pCMV_AncBE4max_P2A_GFP (#112100).

## Source data

Source Data
