# Supplementary material for: Targeting specificity of APOBEC-based cytosine base editor in human iPSCs determined by whole genome sequencing
Source: Nat Commun. 2019 Nov 25;10:5353. doi: 10.1038/s41467-019-13342-8 (PMC6877639; doi:10.1038/s41467-019-13342-8)
Supplement: Supplementary file 1 — Supplementary Information [file 41467_2019_13342_MOESM1_ESM.pdf]

Supplementary Information

Targeting Specificity of APOBEC-based Cytosine Base Editor in Human iPSCs Determined by Whole Genome Sequencing

McGrath, Shin et al.

Supplementary Table 1: Whole genome sequencing statistics

Sample ID	Picard depth	Samtools depth	# Reads	Yield (Mbases)	% Bases >= 30
CL1 Parental	23.4	25.3	323,254,930	96,976	89.95
CL1 Parental	29	30.8	391,963,416	117,589	92.18
CL1-N1	30	30.1	374,477,025	112,343	89.41
CL1-N2	32.7	33.8	435,179,454	130,554	92.47
CL1-N3	29.5	30.2	380,223,124	114,067	92.48
HK31	33.4	34.7	428,082,017	128,425	89.63
HK32M	29.1	29.9	377,001,520	113,100	91.49
HK33M	30.2	32.3	428,018,362	128,406	92.45
HK34	32.4	33.2	412,328,064	123,698	89.59
HK34M	26.2	27.6	348,482,480	104,545	92.52
HK36	30.1	31.8	403,464,291	121,039	91.93
EX1M	30.2	30.2	389,862,750	116,959	91.29
RF23M	29.3	31.7	401,399,024	120,420	92.19
RF24M	23.4	25.5	339,541,224	101,862	92.15
AN21-1	24.8	27.8	429,234,343	128,770	92.44
AN21-2	29.3	29.9	369,661,266	110,898	92.43
ANC-1	28.5	29.2	379,056,876	113,717	92.42
ANC-2	29.5	30.1	391,410,192	117,423	92.76

Supplementary Table 2: Overlap of sequence variants (including on-target editing) identified in each iPSC clone.

	N1	N2	N3	HK31	HK32 M	HK33 M	HK34 M	HK34	HK36	EX1M	RF23 M	RF24 M	AN21 -1	AN21 -2	ANC-1	ANC-2
N1	186	4	4	5	3	2	6	2	3	4	1	1	3	2	1	3
N2	4	171	5	7	2	3	3	5	4	1	1	1	4	0	4	6
N3	4	5	145	4	2	1	4	7	2	4	1	2	5	4	3	4
HK31	5	7	4	164	7	4	7	8	3	4	4	2	5	2	2	7
HK32 M	3	2	2	7	242	4	5	5	4	3	1	3	1	2	0	1
HK33 M	2	3	1	4	4	836	4	4	2	1	0	0	2	2	0	3
HK34 M	6	3	4	7	5	4	113	4	5	4	3	3	4	3	1	5
HK34	2	5	7	8	5	4	4	2300	5	4	2	2	2	3	5	4
HK36	3	4	2	3	4	2	5	5	235	3	0	2	16	18	19	20
EX1M	4	1	4	4	3	1	4	4	3	272	3	3	3	5	3	4
RF23 M	1	1	1	4	1	0	3	2	0	3	599	1	1	3	2	1
RF24 M	1	1	2	2	3	0	3	2	2	3	1	1813	3	2	1	1
AN21-1	3	4	5	5	1	2	4	2	16	3	1	3	7896	494	540	577
AN21-2	2	0	4	2	2	2	3	3	18	5	3	2	494	4605	423	422
ANC-1	1	4	3	2	0	0	1	5	19	3	2	1	540	423	869	495
ANC-2	3	6	4	7	1	3	5	4	20	4	1	1	577	422	495	847

Supplementary Table 3: PCR primers used in this study.

Name	Sequence (5' – 3')	Purpose
AncBE4max-F	CTTCCTACCCTCGTAAAGGTACCGCCACCATGAAACGGACAGCCGAC	Forward primer to amplify the AncBE4max-GFP transgene for construction of the inducible piggyBAC vector XL-AncBE4max
AncBE4max-R	CGCAGGGGAGGTGGTCTACTAGTCAATGGTGATGGTGATGATG	Reverse primer to amplify the AncBE4max-GFP transgene for construction of the inducible piggyBAC vector XL-AncBE4max
HEK3-F	CTTGGCATGAGAAACCTTGG	Forward primer to amplify the genomic region edited by base editor and HEK3 gRNA
HEK3-R	AGCCCCTGTCTAGGAAAAGC	Reverse primer to amplify the genomic region edited by base editor and HEK3 gRNA
EMX1-F	GCCCCTAACCCTATGTAGCC	Forward primer to amplify the genomic region edited by base editor and EMX1 gRNA
EMX1-R	CACCCTAGTCATTGGAGGTGA	Reverse primer to amplify the genomic region edited by base editor and EMX1 gRNA
RNF2-F	ACGTCTCATATGCCCCTTGG	Forward primer to amplify the genomic region edited by base editor and RNF2 gRNA
RNF2-R	ACGTAGGAATTTTGGTGGGACA	Reverse primer to amplify the genomic region edited by base editor and RNF2 gRNA

Note: PCR primers used in off-target validations are listed in Supplementary Data 3.

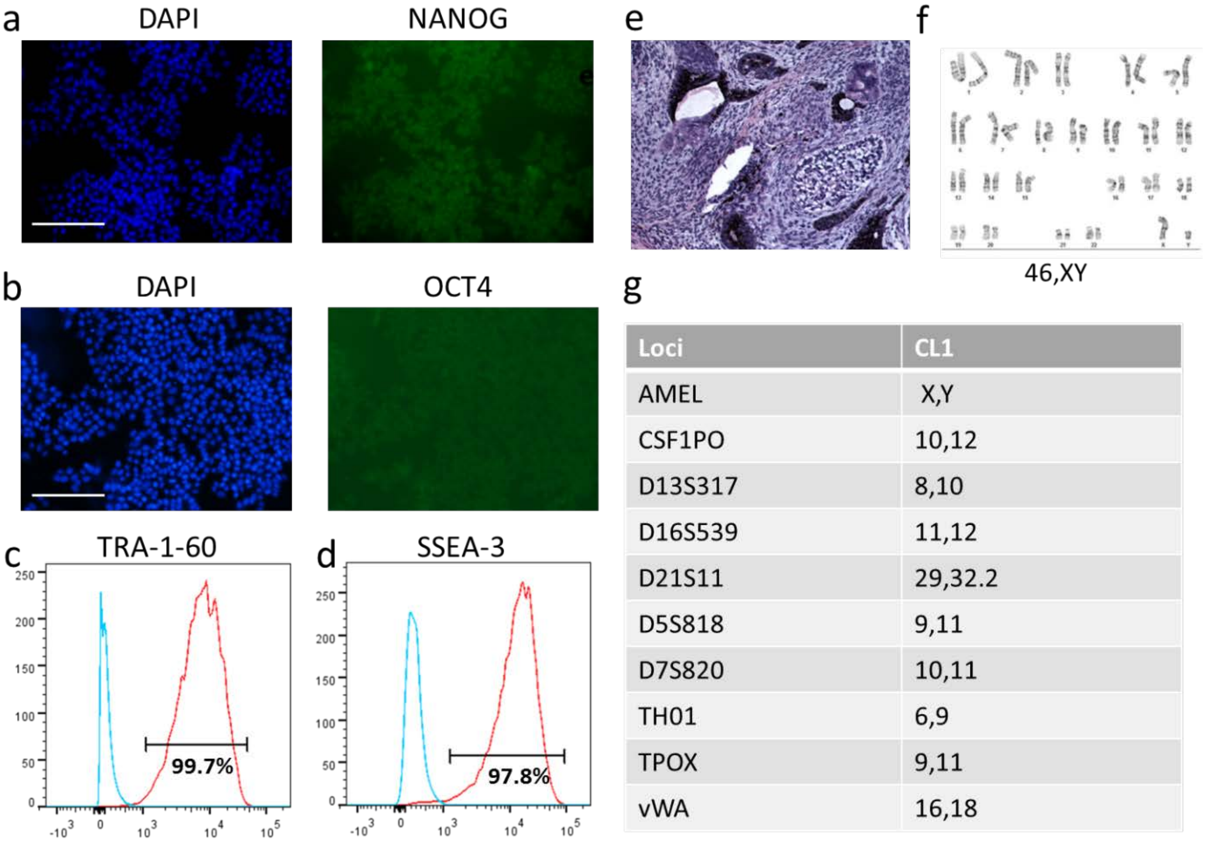

Loci	CL1
AMEL	X,Y
CSF1PO	10,12
D13S317	8,10
D16S539	11,12
D21S11	29,32.2
D5S818	9,11
D7S820	10,11
TH01	6,9
TPOX	9,11
vWA	16,18

Supplementary Figure 1. Characterization of CL1 iPSC line. **a**. Representative images of immunohistochemistry staining of CL1 cells with DAPI (blue) and anit-NANOG antibody (green). Scale bars represent 100µm. **b**. Representative images of immunohistochemistry staining of CL1 cells with DAPI (blue) and anit-OCT4 antibody (green). Scale bars represent 100µm. **c**. Flow cytometry analysis of CL1 cells stained with TRA-1-60 (red) compared to unstained CL1 cells (blue). **d**. Flow cytometry analysis of CL1 cells stained with SSEA-3 (red) compared to unstained CL1 cells (blue). **e**. Representative hematoxylin and eosin staining of teratoma generated from CL1 line. **f**. Karyotype of CL1 line. **g**. Short tandem repeat (STR) profiling of CL1.

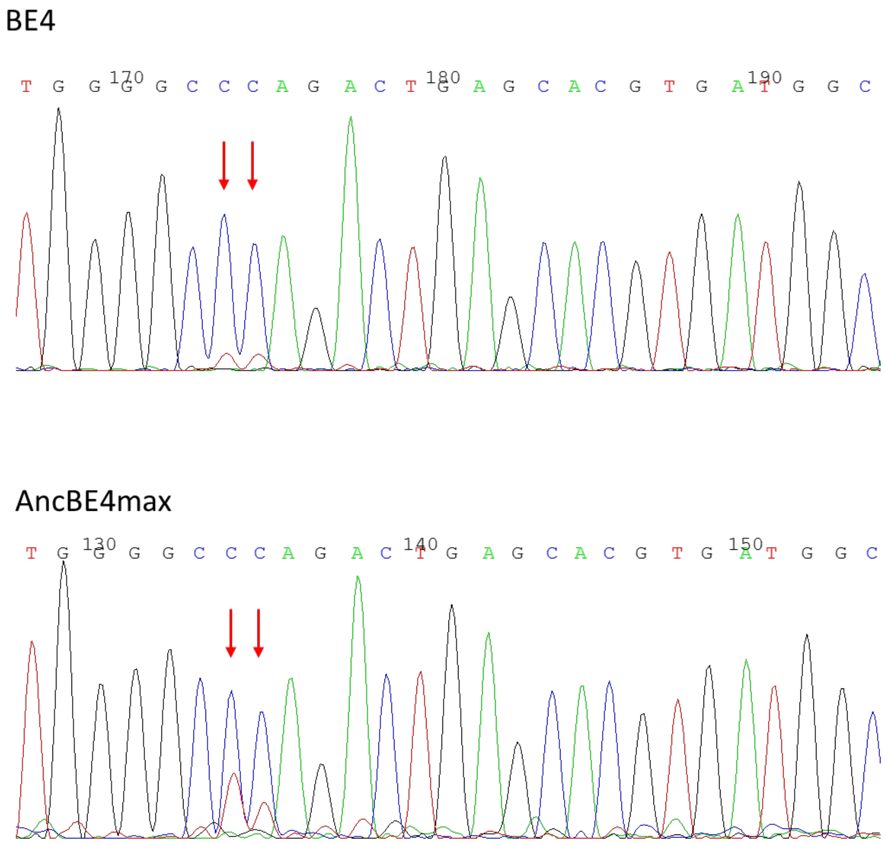

Supplementary Figure 2. Sanger sequencing comparison of iPSCs edited by BE4 or AncBE4max. iPSCs (1 x 10^6)were co-transfected with 3 ug of sgRNA-HEK3-expressing plasmid and 9 ug of either pCMV-BE4 or pCMV-AncBE4max-GFP plasmid. Three days after the transfection, the bulk cell populations were harvested for genomic DNA isolation, followed by PCR amplification as described in Methods. Sanger sequencing results of the PCR products are shown. The red arrows indicate where potential C->T mutations occur.

Sites of C -> T mutations:

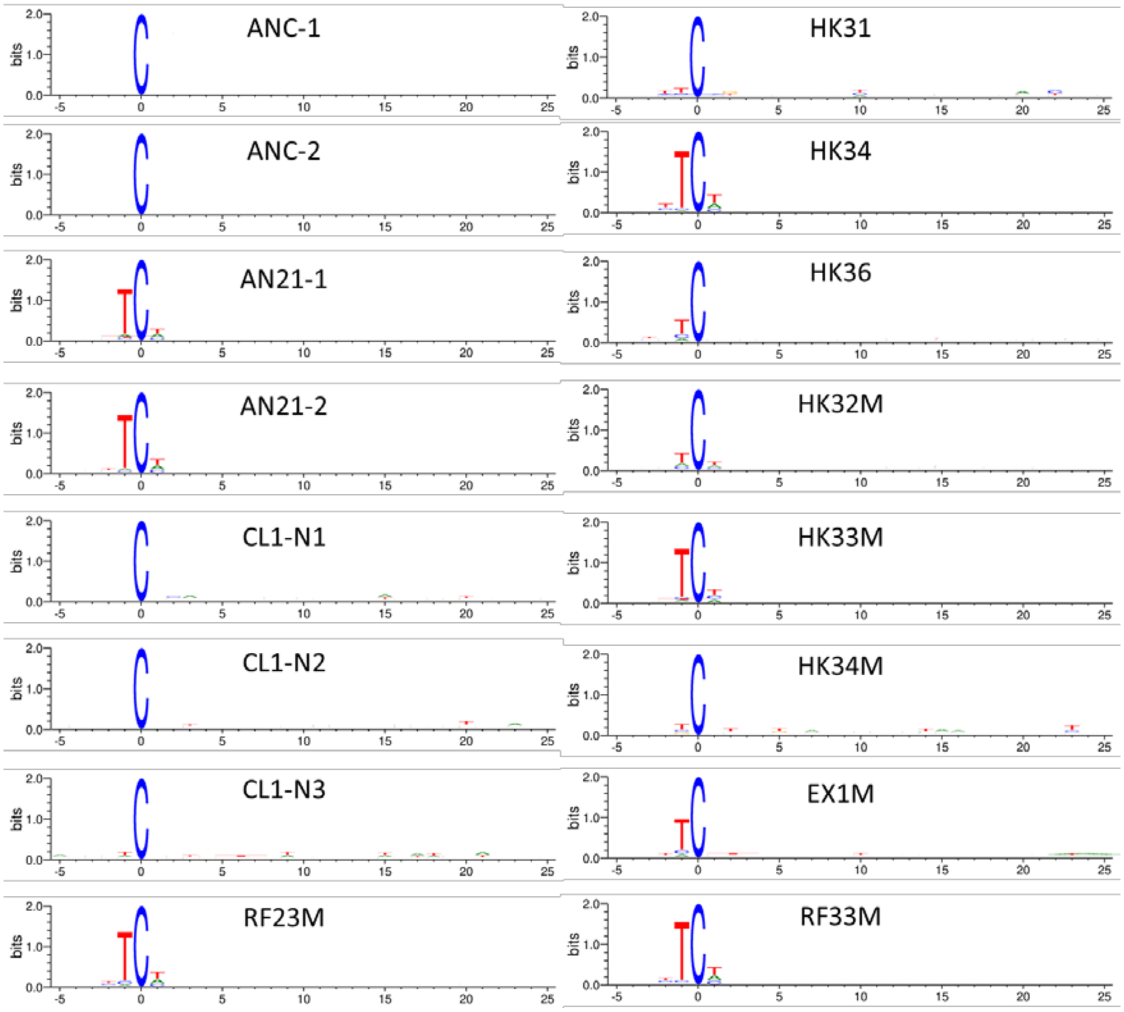

Sites of C -> G mutations:

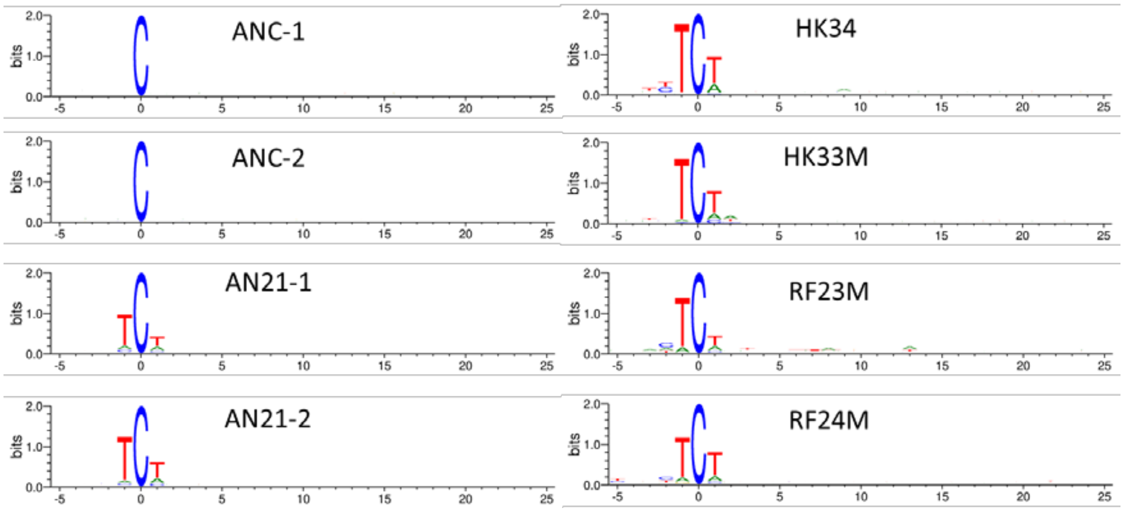

Supplementary Figure 3. Sequence logos of the conserved bases around the C->T and C->G mutations in each iPSC clone. Sequence conservation at positions from -5 to +25 is shown, with the mutated C at position 0.

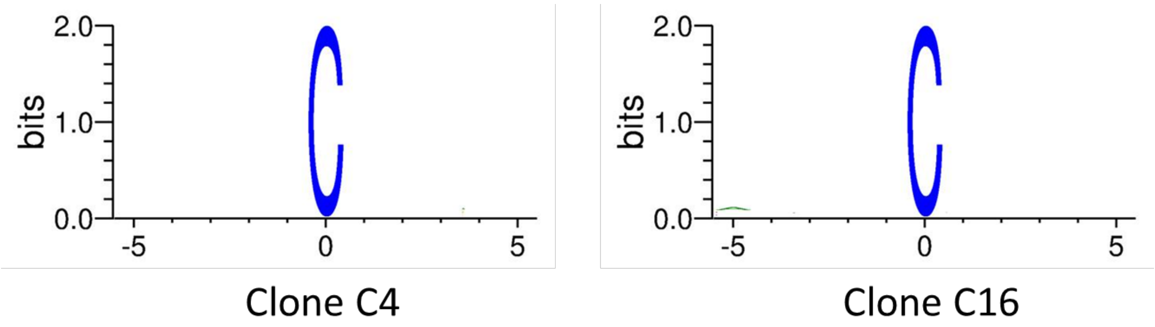

Supplementary Figure 4. Sequence logos of the conserved bases around the C->T mutations in CRISPR/Cas9 edited iPSC clones. Sequence conservation around the mutated C in iPSC Clone C4 and Clone C16, which have been edited by CRISPR/Cas9 at the AAVS1 site (Reference 32). The sequence logos were generated by re-analyzing the WGS data from SRP042612 in NCBI SRA database.

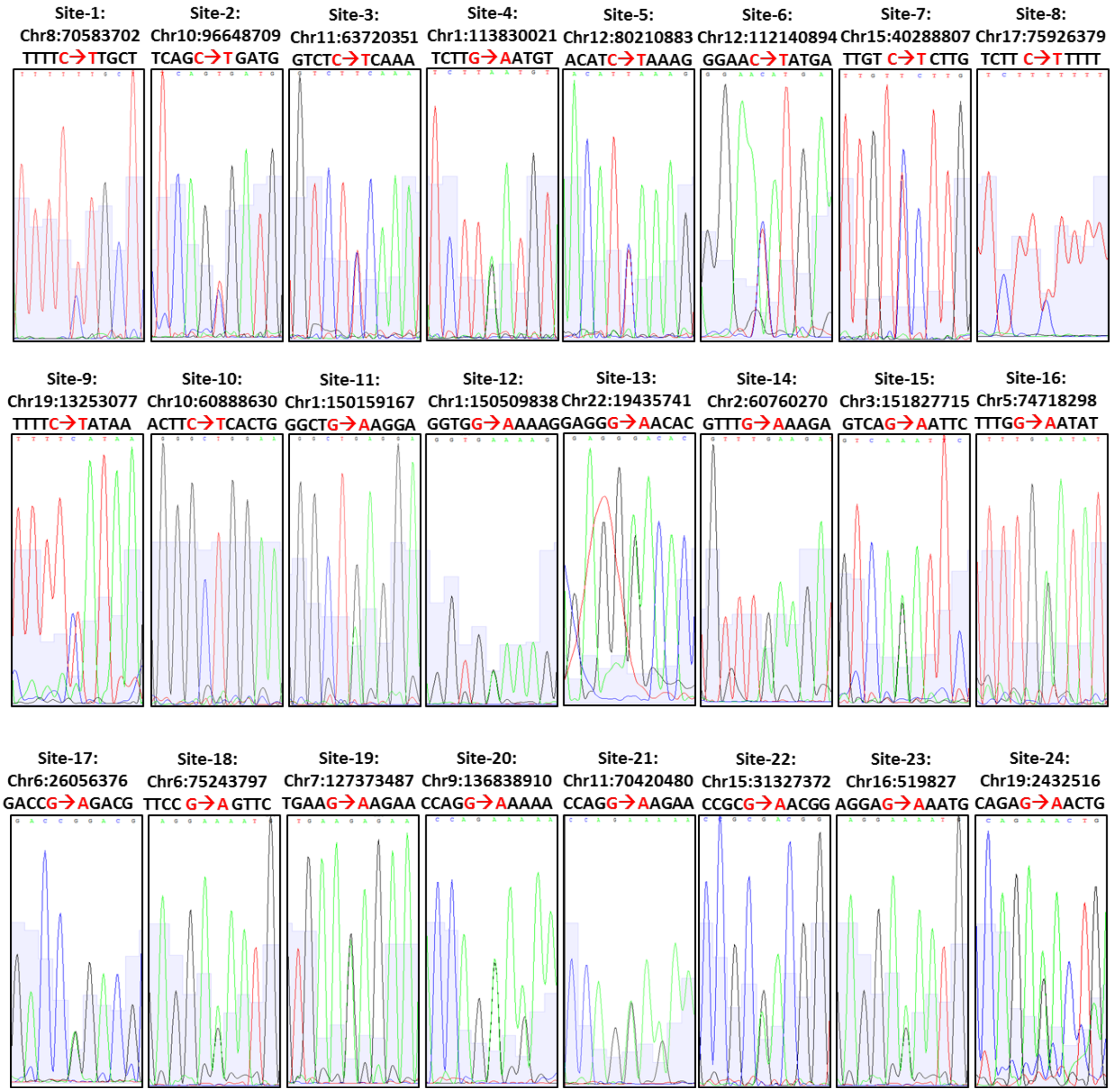

Supplementary Figure 5. Sanger sequencing validation of nonsynonymous exonic SNVs in clone AN21-2. PCR products of the genomic regions containing the WGS-identified mutation sites were sequenced. Electropherogram of each sequencing around the putative mutation site, along with the chromosomal location (based on hg38) are shown. Nucleotide changes are shown in red. Detailed information on the mutation sites and PCR primers can be found in Supplementary Data 3.
