# Supplementary material for: Targeting specificity of APOBEC-based cytosine base editor in human iPSCs determined by whole genome sequencing
Source: Nat Commun. 2019 Nov 25;10:5353. doi: 10.1038/s41467-019-13342-8 (PMC6877639; doi:10.1038/s41467-019-13342-8)
Supplement: Supplementary file 2 — Description of Additional Supplementary Files [file 41467_2019_13342_MOESM2_ESM.pdf]

Title: Supplementary Data 1:
Description: Annotation of all the mutations by wAnnovar.

Title: Supplementary Data 2:
Description: Number of sequence variants identified in base-edited iPSC clones at CRISPR/Cas9 target and in silico-predicted off-target sites.

Title: Supplementary Data 3:
Description: Validation of mutations by amplicon deep sequencing and Sanger sequencing
