# Supplementary material for: Targeting specificity of APOBEC-based cytosine base editor in human iPSCs determined by whole genome sequencing
Source: Nat Commun. 2019 Nov 25;10:5353. doi: 10.1038/s41467-019-13342-8 (PMC6877639; doi:10.1038/s41467-019-13342-8)
Supplement: Supplementary file 6 — Reporting Summary [file 41467_2019_13342_MOESM6_ESM.pdf]

nature research

Corresponding author(s): Zhaohui Ye, Javier Revollo

Last updated by author(s): Oct 12, 2019

Reporting Summary

Nature Research wishes to improve the reproducibility of the work that we publish. This form provides structure for consistency and transparency in reporting. For further information on Nature Research policies, see Authors & Referees and the Editorial Policy Checklist .

Statistics

For all statistical analyses, confirm that the following items are present in the figure legend, table legend, main text, or Methods section.

n/a	Confirmed	
☐	☒	The exact sample size (*n*) for each experimental group/condition, given as a discrete number and unit of measurement
☐	☒	A statement on whether measurements were taken from distinct samples or whether the same sample was measured repeatedly
☐	☒	The statistical test(s) used AND whether they are one- or two-sided<br>*Only common tests should be described solely by name; describe more complex techniques in the Methods section.*
☒	☐	A description of all covariates tested
☒	☐	A description of any assumptions or corrections, such as tests of normality and adjustment for multiple comparisons
☐	☒	A full description of the statistical parameters including central tendency (e.g. means) or other basic estimates (e.g. regression coefficient) AND variation (e.g. standard deviation) or associated estimates of uncertainty (e.g. confidence intervals)
☒	☐	For null hypothesis testing, the test statistic (e.g. *F*, *t*, *r*) with confidence intervals, effect sizes, degrees of freedom and *P* value noted<br>*Give P values as exact values whenever suitable.*
☒	☐	For Bayesian analysis, information on the choice of priors and Markov chain Monte Carlo settings
☒	☐	For hierarchical and complex designs, identification of the appropriate level for tests and full reporting of outcomes
☒	☐	Estimates of effect sizes (e.g. Cohen's *d*, Pearson's *r*), indicating how they were calculated

Our web collection on statistics for biologists contains articles on many of the points above.

Software and code

Policy information about availability of computer code

Data collection	Whole genome sequencing data were collected by Illumina HiSeq2500. Capillary sequencing data were collected by Applied Biosystems 3500xL Genetic Analyzer. Immunofluorescent images were taken by Life Technologies EVOS FL Auto fluorescent microscope.
Data analysis	Sanger sequencing analysis: ApE-A plasmid Editor; NGS data analysis: BWA 0.7.17-r1188, Samtools 1.9, Picardtools, Varscan 2.4.3 (all from the Bioconda repository) and wAnnovar (http://wannovar.wglab.org/) ; Sequence logo generation: Weblogo 3.6.0 (https://weblogo.berkeley.edu/); Microsoft Excel version 1808 (Build 10730.20344 Click-to-Run).

For manuscripts utilizing custom algorithms or software that are central to the research but not yet described in published literature, software must be made available to editors/reviewers. We strongly encourage code deposition in a community repository (e.g. GitHub). See the Nature Research guidelines for submitting code & software for further information.

Data

Policy information about availability of data

All manuscripts must include a data availability statement. This statement should provide the following information, where applicable:

- Accession codes, unique identifiers, or web links for publicly available datasets
- A list of figures that have associated raw data
- A description of any restrictions on data availability

All whole genome sequencing data will be deposited at the SRA repository.

Field-specific reporting

Please select the one below that is the best fit for your research. If you are not sure, read the appropriate sections before making your selection.

Life sciences study design

All studies must disclose on these points even when the disclosure is negative.

Sample size	The samples size is determined based on what have been reported in whole genome sequencing studies of human pluripotent stem cell genome integrity in relationship to reprogramming and genome editing.
Data exclusions	No NGS dataset was excluded in this study. Data from all the sequenced clones were analyzed and presented.
Replication	The effect of editing reagent on genomic sequence was determined by different editing conditions including no guide RNA, guide RNAs targeting different loci, as well as different means of introducing the reagents.
Randomization	Randomization is not required for this study. Necessary controls were included in each experiment to control the covariates.
Blinding	Blinding is not relevant to this study. This is determined based on published studies in the same research field.

Reporting for specific materials, systems and methods

We require information from authors about some types of materials, experimental systems and methods used in many studies. Here, indicate whether each material, system or method listed is relevant to your study. If you are not sure if a list item applies to your research, read the appropriate section before selecting a response.

Materials & experimental systems

n/a	Involved in the study	
☒	☐	Antibodies
☐	☒	Eukaryotic cell lines
☒	☐	Palaeontology
☒	☐	Animals and other organisms
☒	☐	Human research participants
☒	☐	Clinical data

Methods

n/a	Involved in the study	
☒	☐	ChIP-seq
☒	☐	Flow cytometry
☒	☐	MRI-based neuroimaging

Eukaryotic cell lines

Policy information about cell lines

Cell line source(s)	The induced pluripotent stem cell line was generated in our laboratory
Authentication	STR profiling as well as standard pluripotency assays
Mycoplasma contamination	Negativity of mycoplasma contamination was determined by analyzing culture supernatant by MycoAlert reagents.
Commonly misidentified lines (See ICLAC register)	No commonly misidentified lines was used in this study
